# Prevalence and correlates of multiple non-communicable disease risk factors among adults in Zambia: results of the first national STEPS survey in 2017

**DOI:** 10.11604/pamj.2020.37.265.25038

**Published:** 2020-11-24

**Authors:** Supa Pengpid, Karl Peltzer

**Affiliations:** 1 ASEAN Institute for Health Development, Mahidol University, Salaya, Phutthamonthon, Nakhon Pathom, Thailand,; 2 Department of Research Administration and Development, University of Limpopo, Polokwane, South Africa

**Keywords:** Risk factors, non-communicable diseases, prevalence, adults, Zambia

## Abstract

**Introduction:**

the prevalence of Non-Communicable Diseases (NCDs) is increasing in African countries. This study aimed to estimate the prevalence and correlates of multiple NCD risk factors (NCDRF) among the adult population in Zambia

**Methods:**

nationally representative cross-sectional data from 4,302 individuals aged 18-69 years of the “2017 Zambia STEPS survey” were analysed.

**Results:**

the prevalence of insufficient fruit and vegetable consumption was 90.4%, followed by overweight/obesity (24.4%), low physical activity (19.5%), hypertension (18.9%), daily tobacco use (10.7%), sedentary behaviour (8.9%), suicidal behaviour (8.5%), alcohol dependence (7.4%), raised total cholesterol (7.4%), and diabetes (6.2%). The distribution of NCDRF was 41.5% 0-1 NCDRF, 48.2% 2-3, 10.4% 4-10, and 26.7% 3-10 NCDRF. In adjusted ordinal logistic regression analysis, compared to persons aged 18-34 years, individuals aged 50-69 years were 3.58 times (AOR: 3.58, 95% CI: 3.95-4.49) more likely to have a higher number of NCDRF. Women were 24% (AOR: 1.24, 95% CI: 1.03-1.49) more likely than men to have a higher number of NCDRF. Persons living in urban locations were 71% (AOR: 1.74, 95% CI: 1.43-2.16) more likely than persons living in rural locations to have a higher number of NCDRF, and compared to individuals with lower than primary education, persons with more than primary education were 20% (AOR: 0.80, 95% CI: 0.65-0.98) less likely to have a higher number of NCDRF.

**Conclusion:**

more than one in four study participants had three to ten NCDRF and several associated factors were found that can aid to target interventions.

## Introduction

In Zambia 29% of mortality was attributed to non-communicable diseases (NCDs) in 2016 [1]. A high proportion (>80%) of premature deaths from NCDs, such as cardiovascular diseases, diabetes, cancer, and respiratory diseases, occur in low- and middle-income countries [2]. Major behavioural NCD risk factors (NCDRF) increasing the risk of NCD death include tobacco use, unhealthy diet, low physical activity, and hazardous and harmful alcohol use [2]. Considering the increasing trend of NCDs in Africa, it is “crucial to have a careful understanding of the local drivers of NCDs” [3]. Against this backdrop, national data on NCDRF are needed in a lower-middle income Southern African country, Zambia. Some studies in Zambia were subnational (Lusaka urban district) and only focused on specific NCDRF, such as high cholesterol levels (15.8%) [4], obesity (14.2%) [5], diabetes (4.0%) [6] and hypertension (34.8%) [7]. In two rural districts (Kaoma and Kasama) in Zambia, the prevalence of hypertension was 25.8% and 30.3%, respectively [8]. The adult prevalence of diabetes of 3.5% was found in a household survey in five of ten provinces in 2010 in Zambia [9].

In previous national STEPS surveys in three African countries and in Nepal, the prevalence of multiple NCDRF was as follows: 75.8% 4-12 NCDRF (18-69 years in 2015) in Kenya [10], 16.5% 3-7 NCDRF (24-64 years in 2009) in Malawi [11], 17.3% 3-5 NCDRF (18-69 years in 2014) in Uganda [12], and 27.7% 3-8 NCDRF (15-69 years in 2013) in Nepal [13]. Table 1 shows the distribution of individual NCDRF in Kenya, Malawi, Uganda, and Nepal [10-17]. Regarding biological NCDRF, the prevalence of raised total cholesterol ranged from 6.7% in Uganda to 22.6% in Nepal, raised blood ranged from 1.4% in Uganda to 5.6% in Malawi, hypertension ranged from 23.8% in Kenya to 32.0% in Malawi, and general overweight/obesity ranged from 18.1% in Malawi to 27.9% in Kenya. In terms of behavioural NCDRF, the prevalence of inadequate fruit and vegetable intake ranged from 87.8% in Uganda to 98.9% in Nepal, current tobacco smoking ranged from 9.6% in Uganda to 18.5% in Nepal, low physical activity ranged from 3.4% in Nepal to 9.5% in Malawi, and harmful alcohol use (heavy episodic drinking) ranged from 2.0% in Nepal to 16.7% in Uganda (Table 1).

**Table 1:** distribution of individual non-communicable diseases (NCDs) risk factors in four national STEPS surveys

NCD risk factors	STEPS survey country			
	Kenya, 2014 %	Malawi, 2009 %	Uganda, 2014 %	Nepal, 2013 %
Raised total cholesterol	10.1	8.7	6.7	22.6
Raised blood glucose	1.9	5.6	1.4	3.6
Hypertension	23.8	32.9	24.3	25.7
General overweight or obesity	27.9	18.1	23.7	21.4
Inadequate fruit and vegetable intake	94.0	97.5	87.8	98.9
Current tobacco smokers	10.1	14.1	9.6	18.5
Low physical activity	6.5	9.5	4.3	3.4
Harmful alcohol use (Heavy episodic drinking in the past month)	12.7	7.7	16.7	2.0

Factors associated with a higher number of biological and behavioural NCDRF include increasing age [10,13,18-20], women [10], men [13,18], currently married [13], ecological zone [13] or region [12], lower education [13], higher education [18], geolocality [12], higher socioeconomic status [18, 20], and residing in urban areas [18,20]. The study aimed to assess the prevalence and correlates of NCDRF among individuals aged 18-69 years in Zambia.

## Methods

Nationally representative cross-sectional data from the “2017 Zambia STEPS Survey” were analysed [21]. A “multi-stage cluster sampling technique was used to select a nationally representative sample of adults in Zambia aged 18 to 69 years.” [22]. “In the first stage of sampling, Standard Enumeration Areas (SEAs) were selected from each province using a probability proportional to size (PPS), and in the second stage, 15 households in rural SEAs and 20 households in urban SEAs were selected systematically using an appropriate sampling interval based on the number of households in that SEA.”[22]. More information on the sampling strategy and the 2017 Zambia STEPS survey data can be publicly accessed; the survey response rate was 74.3%.” [22]. “The study was approved by the University of Zambia (UNZA) Research Ethics Committee (REC), and written informed consent was obtained from participants.” [22].

### Measures

**NCD outcome variables:** biological NCDRF. Diabetes was classified as “fasting plasma glucose levels =7.0 mmol/L, and/or currently taking insulin or oral hypoglycemic drugs”; raised total cholesterol (TC) as “fasting TC =5.0 mmol/L or currently on medication for raised cholesterol”; hypertension as “systolic BP =140 mm Hg and/or diastolic BP =90 mm Hg or currently on antihypertensive medication”; and measured Body Mass Index (”25-29.9 kg/m2 overweight and =30 kg/m2 obesity”) [22].

Behavioural NCDRF consisted of insufficient fruit and vegetable consumption (<5 servings/day), low physical activity, and sedentary behaviour (=8 hours/day) based on the “Global Physical Activity Questionnaire”, daily tobacco use, and alcohol dependence (defined as =4 scores on item 4-6 of the “Alcohol Use Disorder Identification Test=AUDIT” [22]. Suicidal behaviour was based on three questions on suicidal ideation, plans, and/or attempts in the past year [22]. Sociodemographic information included marital status, age, sex, highest educational level, work status, ethnic affiliation, and geolocality [22].

**Data analysis:** statistical procedures were done with “STATA software version 15.0 (Stata Corporation, College Station, Texas, USA),” taking the multistage sampling design and data weighting into account [22]. The total number of ten NCDRF were grouped into 1=0-1, 2=2-3, and 3=4-10 NCDRF and described with bar graphs and frequency tabulations. Unadjusted and adjusted ordinal logistic regressions were utilized to assess predictors of the number of NCDRF (0-1, 2-3, and 4-10). Co-variates were selected based on a previous literature review [10,13,18-20]. Only complete cases were included in the analysis, and p<0.05 was set as significant.

## Results

**Characteristics of the sample and NCDRF:** the study population included 4,302 individuals aged 18-69 years (31 years median age, IQR 23-41). Almost half of the study participants (48.7%) were men, 59.1% were married or cohabiting, 28.7% were unemployed, 71.1% had primary or more education, 32.8% were Bemba and 48.8% resided in urban areas. The prevalence distribution of individual biological NCDRF was 7.4% raised total cholesterol, 6.2% diabetes, 24.4% overweight/obesity, and 18.9% hypertension. The prevalence distribution of individual behavioural NCDRF was 90.4% insufficient fruit and vegetable consumption, 19.5% low physical activity, 10.7% daily tobacco use, 8.9% sedentary behaviour, 8.5% suicidal behaviour, and 7.4% alcohol dependence. The prevalence of overweight/obesity, low physical activity, raised total cholesterol, and suicidal behaviour was significantly higher in women than in men, and the prevalence of daily tobacco use and alcohol dependence was significantly higher in men than in women (Table 1, Table 2).

**Table 2:** sample characteristics of survey participants aged 18-69 years in Zambia, 2017 (N=4302)

Variable	Sample	All	Male	Female
	Unweighted number	Weighted % (95% CI)	Weighted % (95% CI)	Weighted % (95% CI)
**Socio-demographics**				
Age in years				
18-34	2182	59.6 (57.7, 61.4)	60.1 (56.7, 63.3)	59.1 (56.7, 61.5)
35-49	1288	28.0 (26.3, 29.7)	28.4 (25.4, 31.5)	27.6 (25.8, 29.6)
50-69	832	12.4 (11.3, 13.6)	11.6 (9.9, 13.5)	13.2 (11.8, 14.9)
Sex				
Male	1614	48.7 (47.0, 50.4)	---	---
Female	2688	51.3 (49.6, 53.0)		
Highest level of education				
<Primary	1546	28.8 (26.5, 31.2)	22.6 (20.0, 25.3)	34.8 (31.8, 37.8)
Primary	1036	23.0 (21.2, 25.0)	22.3 (19.6, 25.3)	23.7 (21.7, 25.8)
>Primary	1717	48.1 (45.2, 51.1)	55.1 (51.4, 58.7)	41.5 (38.1, 45.0)
Marital status				
Married/cohabiting	2627	59.1 (57.0, 61.2)	60.8 (57.8, 63,7)	57.5 (55.0, 60.0)
Widowed/divorced/separated/never married	1665	40.9 (38.8, 43.0)	39.2 (36.3, 42.2)	42.5 (40.0, 45.0)
Employment status				
Employed	2147	50.4 (47.9, 52.9)	60.1 (56.7, 63.5)	41.1 (38.4, 43.9)
Nonpaid	852	20.9 (18.9, 23.0)	16.0 (13.5, 18.9)	25.5 (23.0, 28.2)
Unemployed	1296	28.7 (26.0, 31.6)	23.8 (20.7, 27.3)	33.4 (30.2, 36.7)
Geolocality				
Rural	2660	51.2 (48.3, 54.2)	53.8 (50.0, 57.6)	48.8 (45.7, 51.9)
Urban	1642	48.8 (45.8, 51.7)	46.2 (42.4, 50.0)	51.1 (48.1, 54.3)
Ethnic group				
Bemba	1260	32.8 (29.0, 36.8)	33.6 (29.0, 38.5)	32.0 (28.2, 36.0)
Tonga	1230	33.7 (30.2, 37.3)	33.5 (29.3, 38.0)	33.9 (30.3, 37.7)
Other	1478	33.5 (30.0, 37.3)	32.9 (28.6, 37.4)	34.2 (30.3, 38.2)
**Non-communicable diseases risk factors**				
Fruit/vegetable consumption (<5 servings/day)	3604	90.4 (88.4, 92.1)	90.0 (87.5, 92.0)	90.8 (88.6, 92.7)
Low physical activity	869	19.5 (17.5, 21.7)	12.5 (10.5, 14.9)	26.2 (23.4, 29.2)
Sedentary behaviour (≥8 hours/day)	408	8.9 (7.6, 10.5)	8.0 (6.4, 9.9)	9.9 (8.1, 12.0)
Daily tobacco use	454	10.7 (9.5, 12.0)	17.7 (15.6, 20.0)	4.0 (3.2, 5.0)
Alcohol dependence	242	7.4 (6.3, 8.6)	12.0 (10.1, 14.3)	3.0 (2.2, 4.1)
Diabetes	266	6.2 (5.2, 7.3)	6.0 (4.5, 7.8)	6.4 (5.3, 7.8)
Hypertension	852	18.9 (17.4, 20.5)	20.5 (18.2, 23.1)	17.4 (15.7, 19.3)
Raised total cholesterol	344	7.4 (6.4, 8.5)	5.0 (3.8, 6.5)	9.7 (8.2, 11.4)
General overweight/obesity	1020	24.4 (22.5, 26.3)	16.2 (14.0, 18.7)	32.6 (30.2, 35.1)
Suicidal behaviour	368	8.5 (7.5, 9.7)	5.8 (4.6, 7.4)	11.1 (9.6, 12.8)
CI=Confidence Interval				

**Figure 1:**
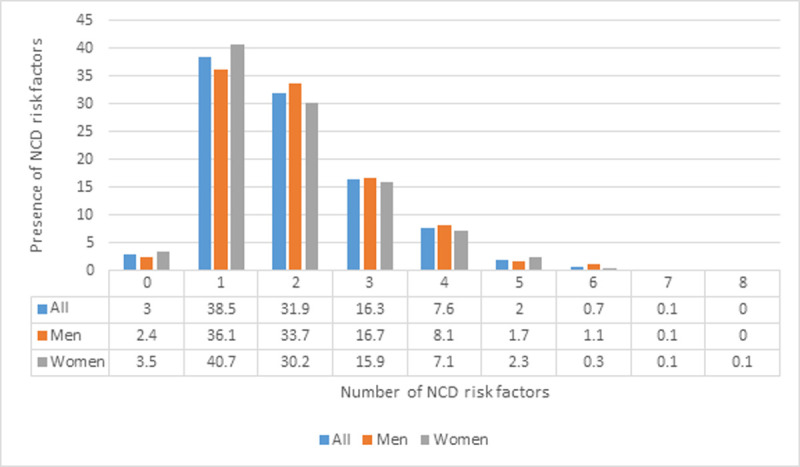
frequency of non-communicable diseases risk factors among adults in Zambia

**Frequency distribution of NCDRF:** the prevalence of having no NCDRF was 3.0%, one 38.5%, two 31.9%, three 16.3%, four 7.6%, five 2.0%, six 0.7%, seven 0.1% and 8-10 risk factors zero percent (Figure 1). Overall, 41.5% of the participants had 0-1 NCD risk factor, 48.2% 2-3 risk factors, 10.4% 4-10, and 26.7% three or more NCDRF. A higher number of NCDRF increased with age, urban residence, and female sex, and among men who were never married, separated, divorced, or widowed (Table 3).

**Table 3:** distribution of non-communicable diseases (NCDs) risk factor counts among individuals aged 18-69 years in Zambia, 2017

Variable	All			Male			Female		
	NCD risk factors			NCD risk factors			NCD risk factors		
	n	n	n	n	n	n	n	n	n
	0-1	2-3	4-10	0-1	2-3	4-10	0-1	2-3	4-10
	%	%	%	%	%	%	%	%	%
All	41.5	48.2	10.4	44.2	46.1	9.8	38.6	50.4	11.0
**Age in years**									
18-34	48.9	44.7	6.3	52.4	41.7	5.9	45.1	48.0	6.8
35-49	32.9	54.5	12.6	32.7	53.4	13.9	33.0	55.9	11.1
50-69	24.3	50.3	25.4	28.2	50.8	21.0	21.1	49.9	29.0
p-value	<0.001			<0.001			<0.001		
**Sex**									
Male	44.2	46.1	9.8						
Female	38.6	50.4	11.0						
p-value	<0.001								
**Highest level of education**									
<Primary	38.5	49.2	12.3	38.1	50.0	11.9	38.9	48.6	12.5
Primary	45.8	44.6	9.6	49.1	41.1	10.0	42.5	48.2	9.2
>Primary	41.3	49.1	9.6	44.9	46.3	8.8	36.3	52.9	10.7
p-value	0.343			0.260			0.433		
**Marital status**									
Married/cohabiting	39.2	50.0	10.8	38.5	50.0	11.5	40.1	50.0	9.9
Widowed/divorced/separated/never married	44.4	45.8	9.8	52.6	40.2	7.2	36.6	51.1	12.2
p-value	0.454			0.008			0.178		
**Work status**									
Employed	36.9	51.8	11.2	38.1	51.1	10.8	35.1	53.1	11.9
Nonpaid	47.8	42.9	9.3	53.9	39.7	6.4	43.8	45.0	11.2
Unemployed	44.7	45.7	9.6	53.2	37.3	9.5	38.2	52.1	9.7
p-value	0.093			0.050			0.349		
**Geolocality**									
Rural	44.9	47.2	7.9	45.0	46.5	8.5	44.8	48.1	7.1
Urban	37.9	49.2	13.0	43.1	45.5	11.4	33.3	52.4	14.3
p-value	<0.001			<0.001			<0.001		
**Ethnic group**									
Bemba	40.9	48.7	10.4	45.3	46.9	7.8	36.2	50.6	13.2
Tonga	39.9	49.7	10.3	40.3	49.1	10.6	39.5	50.5	10.0
Other	45.0	45.0	9.9	47.4	43.2	9.4	42.6	46.9	10.5
p-value	0.151			0.148			0.608		

**Associations with the number of NCDRF:** Table 4 shows the univariate associations with NCDRF counts. In the adjusted ordinal logistic regression analysis, compared to persons aged 18-34 years, individuals aged 50-69 years were 3.58 times (AOR: 3.58, 95% CI: 3.95-4.49) more likely to have a higher number of NCDRF. Women were 24% (AOR: 1.24, 95% CI: 1.03-1.49) more likely than men to have a higher number of NCDRF. Persons living in urban locations were 71% (AOR: 1.74, 95% CI: 1.43-2.16) more likely than persons living in rural locations to have a higher number of NCDRF, and compared to individuals with lower than primary education, persons with more than primary education were 20% (AOR: 0.80, 95% CI: 0.65-0.98) less likely to have a higher number of NCDRF (Table 5).

**Table 4:** univariate ordinal logistic regression with non-communicable diseases risk factor counts among individuals aged 18-69 years in Zambia, 2017

Variable	All	Men	Women
	0-1 vs. 2-3 & 4-10 risk factors	0-1 vs. 2-3 & 4-10 risk factors	0-1 vs. 2-3 & 4-10 risk factors
	COR (95% CI)	COR (95% CI)	COR (95% CI)
**Age in years**			
18-34	1 (Reference)	1 (Reference)	1 (Reference)
35-49	1.96 (1.58, 2.44)^***^	2.30 (1.65, 3.21)^***^	1.65 (1.27, 2.14)^***^
50-69	3.72 (2.80, 4.94)^***^	3.22 (2.10, 4.94)^***^	4.12 (2.82, 6.02)^***^
**Sex**			
Male	1 (Reference)	---	---
Female	1.23 (1.01, 1.51)^*^		
**Highest level of education**			
<Primary	1 (Reference)	1 (Reference)	1 (Reference)
Primary	0.74 (0.57, 0.96)^*^	0.66 (0.44, 0.99)^*^	0.82 (0.59, 1.15)
>Primary	0.86 (0.69, 1.08)	0.75 (0.53, 1.04)	1.04 (0.77, 1.41)
**Marital status**			
Married/cohabiting	1 (Reference)	1 (Reference)	1 (Reference)
Widowed/divorced/separated/never married	0.91 (0.77, 1.07)	0.67 (0.51, 0.87)^**^	1.19 (0.96, 1.47)
**Work status**			
Employed	1 (Reference)	1 (Reference)	1 (Reference)
Nonpaid	0.78 (0.62, 0.99)^*^	0.64 (0.43, 0.96)^*^	0.83 (0.65, 1.05)
Unemployed	0.90 (0.74, 1.09)	0.75 (0.56, 1.01)	0.96 (0.76, 1.22)
**Geolocality**			
Rural	1 (Reference)	1 (Reference)	1 (Reference)
Urban	1.50 (1.26, 1.78)^***^	1.24 (0.95, 1.63)	1.76 (1.43, 2.18)^***^
**Ethnic group**			
Bemba	1 (Reference)	1 (Reference)	1 (Reference)
Tonga	1.03 (0.79, 1.34)	1.25 (0.84, 1.86)	0.84 (0.60, 1.16)
Other	0.86 (0.68, 1.09)	0.96 (0.68, 1.37)	0.76 (0.56, 1.04)

*** p<0.001; ^**^p<0.01; ^*^P<0.05; COR=Crude Odds Ratio; CI=Confidence Interval

**Table 5:** multivariable ordinal logistic regression with non-communicable diseases risk factor counts among individuals aged 18-69 years in Zambia, 2017

Variable	All	Men	Women
	0-1 vs. 2-3 & 4-10 risk factors	0-1 vs. 2-3 & 4-10 risk factors	0-1 vs. 2-3 & 4-10 risk factors
	AOR (95% CI)	AOR (95% CI)	AOR (95% CI)
**Age in years**			
18-34	1 (Reference)	1 (Reference)	1 (Reference)
35-49	1.72 (1.43, 2.08)^***^	1.53 (1.12, 2.09)^**^	1.97 (1.59, 2.46)^***^
50-69	3.58 (2.85, 4.49)^***^	2.86 (1.99, 4.11)^***^	4.46 (3.29, 6.05)^***^
**Sex**			
Male	1 (Reference)	---	---
Female	1.24 (1.03, 1.49)^*^		
**Highest level of education**			
<Primary	1 (Reference)	1 (Reference)	1 (Reference)
Primary	0.82 (0.67, 1.02)^*^	0.65 (0.46, 0.91)^*^	0.99 (0.75, 1.30)
>Primary	0.80 (0.65, 0.98)^*^	0.66 (0.49, 0.88)^**^	0.94 (0.71, 1.24)
**Marital status**			
Married/cohabiting	1 (Reference)	1 (Reference)	1 (Reference)
Widowed/divorced/separated/never married	1.03 (0.86, 1.22)	0.91 (0.65, 1.26)	1.08 (0.87, 1.34)
**Work status**			
Employed	1 (Reference)	1 (Reference)	1 (Reference)
Nonpaid	0.85 (0.66, 1.08)	0.88 (0.56, 1.40)	0.87 (0.67, 1.11)
Unemployed	0.98 (0.79, 1.20)	0.85 (0.62, 1.18)	1.12 (0.87, 1.45)
**Geolocality**			
Rural	1 (Reference)	1 (Reference)	1 (Reference)
Urban	1.76 (1.43, 2.16)^***^	1.49 (1.10, 2.02)^**^	2.13 (1.64, 2.77)^***^
**Ethnic group**			
Bemba	1 (Reference)	1 (Reference)	1 (Reference)
Tonga	1.19 (0.98, 1.44)	1.33 (0.98, 1.81)	1.04 (0.79, 1.37)
Other	0.97 (0.78, 1.20)	0.97 (0.70, 1.35)	0.96 (0.74, 1.25)

*** p<0.001; ^**^p<0.01; ^*^P<0.05; AOR=Adjusted Odds Ratio; CI=Confidence Interval

## Discussion

The nationally representative 2017 Zambia STEPS survey among individuals aged 18-69 years found a high prevalence of 3-10 NCDRF (26.7%), which was similar to Nepal (27.7% 3-8 NCDRF [13], lower than in Kenya (75.8% 4-12 NCDRF [10], but higher than in Malawi (16.5% 3-7 NCDRF) [11], and Uganda (17.3% 3-5 NCDRF) [12]. The high clustering of NCDRF in this survey increases the risk of NCDs in the adult population in Zambia. Consistent with previous research [10,13-20], this survey found that increasing age, female sex, lower education, and urban residence were associated with a higher number of NCDRF. The promotion of early screening for NCDRF, targeting women, urban residents, and those with lower education, may help the prevention of the development of NCDs in Zambia. Unlike some previous research [13,20], this study did not find any significant association between marital status, employment status, ethnic group, and multiple NCDRF.

The four most prevalent individual NCDRF in this study were insufficient fruit and vegetable consumption (90.4%), overweight/obesity (24.4%), low physical activity (19.5%), and hypertension (18.9%). Similar proportions of individual NCD risk behaviours were found in the STEPS surveys in 2014 in Kenya [10] and in 2013 in Nepal [13]. The prevalence of hypertension (18.9%) in this study was lower than in previous local surveys in Zambia, 34.8% in the urban Lusaka district [7], and 25.5%-30.3% in two rural districts in Zambia [8]. The prevalence of overweight/obesity (24.4%) in this study was higher than in the urban Lusaka district study (14.2%) [5], and the prevalence of insufficient fruit/vegetable consumption (90.4%) was higher than in the 2003 Zambia World Health Survey (77.7%) [23], and the prevalence of low physical activity (19.5%) in this study was similar to data from the 2003 Zambia World Health Survey (23.3%) [23].

The prevalence of daily tobacco use (10.7%) and alcohol dependence (7.4%) in this survey was similar to the 2009 Malawi STEPS survey (14.1% smokers, and 7.7% excessive drinkers) [11,15], and the 2014 Kenya STEPS survey (10.1% smokers, and 12.7% harmful alcohol users) [10,14]. The proportions of daily tobacco use and alcohol dependence were similar to results from the 2003 Zambia World Health Survey (14.1% current smoking and 7.4% heavy episodic drinking) [23]. The increase of exercise taxes and prices on tobacco products and alcoholic beverages has been recommended in Zambia [22]. The prevalence of diabetes (6.2%) and raised total cholesterol (7.4%) in this study was similar to the 2009 Malawi STEPS survey (5.6% diabetes and 8.7% raised cholesterol) [11,15], but lower than in the 2014 Kenya STEPS survey in terms of high total cholesterol (10.1%), and higher in terms of diabetes (1.9%) [10,14]. Compared to the prevalence of raised total cholesterol (15.8%) in the Lusaka urban district STEPS survey [4], the prevalence of raised total cholesterol was lower in this study (7.4%), and the prevalence of diabetes (6.2%) was higher in this study than in the Lusaka urban district study (4.0%) [6], and the large community-based study in 2010 in Zambia (3.5%) [9].

Specific NCDRF differed by sex in this study, with substance being higher in men, and overweight/obesity, low physical activity, raised total cholesterol, and suicidal behaviour being higher in women. Similar sex differences in the prevalence of substance use, obesity, and raised total cholesterol were also found in the 2009 Malawi and 2014 Kenya STEPS surveys [10,11]. It is important to take these sex differences into account when designing NCD health promotion activities [11].

Study limitations include the cross-sectional design of the study, which prevents from causative inferences, and the questionnaire interview relying on self-report of the data. Some study variables, such as household income, could not be included in the analysis due to too many missing values.

## Conclusion

In this national community-based 2017 STEPS survey among adults in Zambia, more than one in four study participants had three to ten NCDRF. Several factors associated with NCDRF counts were identified, including increasing age, female sex, residing in urban areas, and lower education that can be targeted in interventions to address multiple NCDRF in the Zambian population. Taking the clustering nature of NCDRF into account, interventions should be targeting multiple, in particular modifiable, NCDRF.

### What is known about this topic

- Some subregional studies in Zambia report the prevalence of individual NCD risk factors;
- Some African countries report on the national prevalence of individual NCD risk factors;
- Some African countries, such as Kenya, Malawi, and Uganda, report on the national prevalence of multiple NCD risk factors.

### What this study adds

- More than one in four persons in Zambia had three or more NCD risk factors;
- Older age, female sex, rural residence, and lower education increased the odds for an increasing number of NCD risk factors in the Zambian population;
- Taking the clustering nature of NCD risk factors into account, interventions should be targeting multiple, in particular modifiable, NCD risk factors.
